# The ‘land of fires’: epidemiological research and public health policy during the waste crisis in Campania, Italy

**DOI:** 10.1016/j.heliyon.2022.e12331

**Published:** 2022-12-15

**Authors:** Piero Alberti

**Affiliations:** University of Oxford, Medical Sciences Division, UK

**Keywords:** Biomonitoring, Campania, Cancer mortality, Environmental exposure, Epidemiological risk, Illegal waste dumping, Indigenous knowledge, Public health, Precautionary principle, Toxic waste

## Abstract

The region of Campania, South Italy rose to prominence in the mid-2000s due to the illegal disposal of waste by the Camorra during the local waste management crisis. Several lines of evidence have identified a link between exposure to toxic waste and adverse health outcomes in the local populations. Critically, studies since 2017 have strongly suggested that this link is causal in nature. The uncertainty of evidence polarised the Italian epidemiological community and partly undermined the precautionary principle in public health policy, leading to years of delay in the deployment of appropriate interventions. The crisis also sparked concerns about pollution of soil, water, and agricultural products. The contrast between political responses and protests from local communities shows analogies with environmental emergencies of a larger scale. Beyond law enforcement actions to prosecute illegal waste disposal activity, future mitigation of risks for affected populations will require coordinated efforts in environmental policy (land reclamation, improved waste management) and public health (i.e. extensive epidemiological surveillance, screening and prevention programs). By summarising evidence over the last two decades, this review aims to construct a cohesive interdisciplinary narrative of the events in the Campanian waste crisis.

## 1 Introduction

The ‘land of fires’ (*terra dei fuochi*)^1^ is an area in the Southern Italian region of Campania that rose to prominence in the mid-2000s due to the local disposal of toxic waste by organised crime syndicates belonging to the Camorra, often defined as ‘eco-mafia’ [1]. According to the Italian Ministry of Agriculture, the ‘land of fires’ is home to around 3 million inhabitants over the area of 90 municipalities, 56 of which are in the Metropolitan City of Naples and 34 in the Province of Caserta [2, 3, 4]. Studies in the past 20 years have shown a correlation between waste disposal in the areas and significant increases in incidence and mortality from cancer and other diseases, with the most recent evidence suggesting a causal link between exposure to toxic waste and morbidity and mortality patterns in the ‘land of fires’. By summarising evidence from a limited subset of available literature, this article provides an interdisciplinary historical summary of a long-lasting environmental and public health crisis.

**Figure 1:**
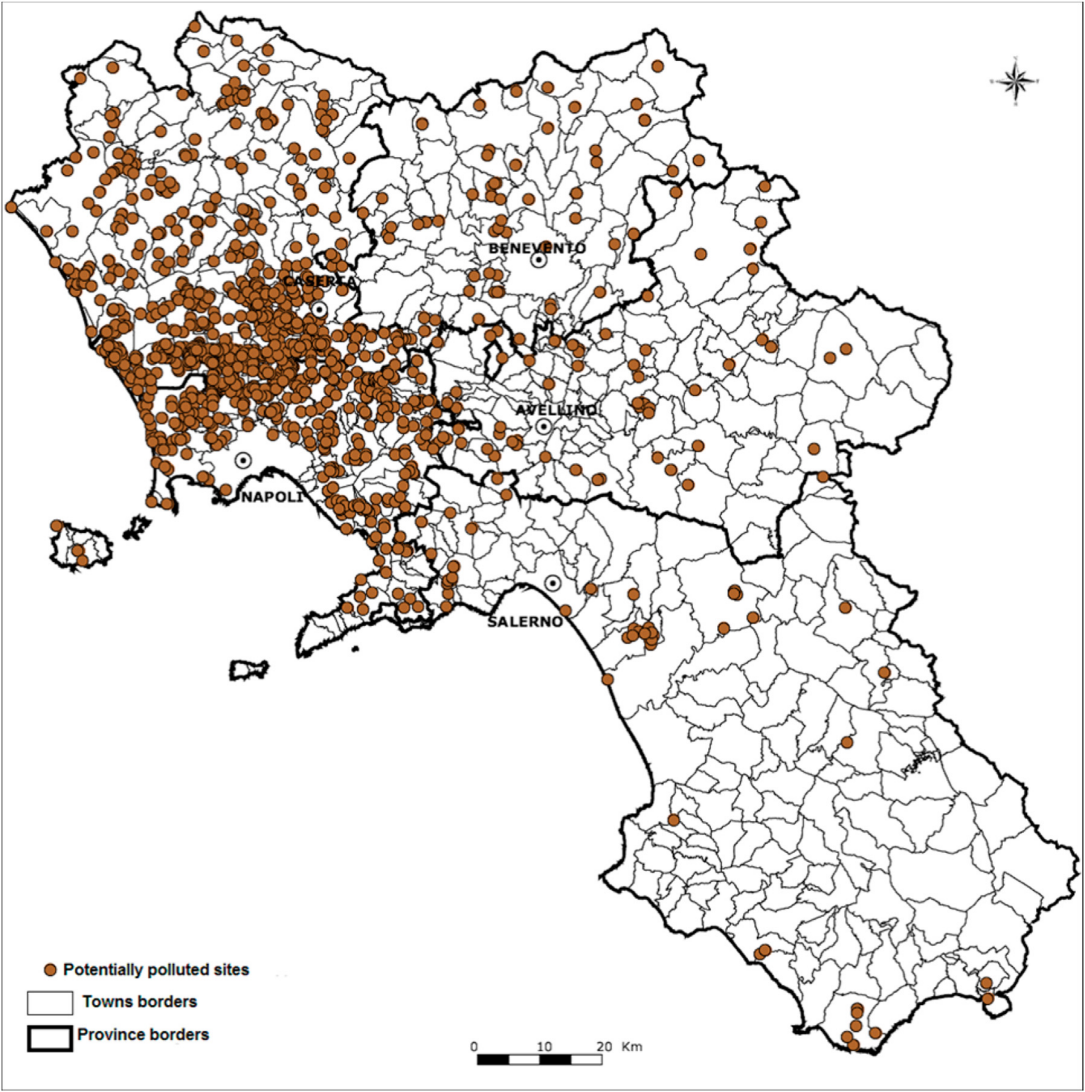
Hazardous waste disposal sites in the Campanian waste crisis [2007-8]. The density of contaminated waste sites is clearly highest in the Naples and Caserta provinces. *Adapted from*: Mazza A et al. Heavy Environmental Pressure in Campania and Other Italian Regions: A Short Review of Available Evidence. Int J Environ Res Public Health. 2018;15(1).

Whilst the eco-mafia's monopoly on waste management in Campania was first denounced by environmentalist organisations like Legambiente in the late 1980s, it was investigations by the Naples Police in 1994-6 that revealed the role of various Camorra clans in contacting industries in Northern Italy to offer low-cost disposal of their waste products, which were then trafficked to Campania and dumped or burned in unregulated landfills [5, 6, 7]. While illegal international trafficking of waste is common, this type of intra-national waste trafficking appears to be globally unique. According to statements by repented Camorra bosses (*pentiti*), trafficking in the 1980s–90s had turned the Naples and Caserta territory into an open-air landfill for Italy's toxic urban and industrial waste including heavy metals, sewage sludge, battery acids, asbestos, and radioactive waste. In the years 1990–2005, the Camorra is estimated to have trafficked ∼14 million tonnes of waste for a €44 billion profit [7]. The ecomafia's activity peaked in 2007-08, when toxic waste fires lit by the Camorra could be camouflaged among fires of urban waste lit by ordinary citizens after the collapse of the regional landfill infrastructure (Figure 1) [8,9].

### 2 The epidemiological debate: correlation and causation in the Campanian case

While the waste crisis had been declared a national emergency since Campanian landfills were saturated in 1994, evidence for adverse health effects came with later studies by public health authorities. In 2001, a report by the National Institute of Health (ISS) found that the distribution of childhood mortality in the Province of Caserta was related to that of local landfills [10, 11]. A study by the Italian Epidemiology Association (AIE) in 2004 then found a significant excess risk of mortality from different cancer types, cardiovascular disease and diabetes in Campanian municipalities with high intensity of toxic waste disposal sites relative to the rest of the region [12]. While not suggesting a causal link, these early studies made recommendations that would be central in the later epidemiological debate, calling for land reclamation efforts and stressing the need for institutional transparency and shared decision-making in affected communities.

Studies aiming to delineate a causal link between exposure to toxic waste in the ‘land of fires’ and morbidity patterns have proven difficult due to three key factors. Firstly, the multifactorial and protracted pathogenesis of most cancer types complicates attempts to define a cause-effect relationship between exposure to environmental pollutants and incidence of disease. Secondly, the population of interest is very large and characterised by highly heterogenous environmental exposures that influence the epidemiology of cancer, including lifestyle factors, occupational exposures, socioeconomic status, access to care (screening, prevention, specialist services) and air pollution from urban traffic and industries in the area [13]. Finally, the nature, quantity and distribution of toxic pollutants to which the population was exposed is mostly unknown due to the unregulated nature of their disposal, as is the actual duration of the exposures themselves.

The alarm bell that brought the possibility of a public health crisis in Campania to international attention was actually first rung in the UK, when a report by National Research Council (CNR) member Alfredo Mazza in *The Lancet Oncology* showed that mortality from several types of cancer, in particular liver cancer and leukaemias, was elevated around the towns of Acerra, Nola and Marigliano – the ‘triangle of death’ – relative to neighbouring areas (Table 1) [14].

**Table 1:** Standardised death rates per 100,000 population. *Adapted from:* Senior K, Mazza A. Italian "Triangle of death" linked to waste crisis. Lancet Oncol. 2004;5(9):525-7.

	Campania	East Naples (ASL NA4)	Triangle of death (D73)
All	305.6 (M)	301.8 (M)	321.7 (M)
	195.7 (F)	177.5 (F)	189.7 (F)
Colorectal	26.4 (M)	27.2 (M)	23.6 (M)
	26.4 (F)	21.2 (F)	29.0 (F)
Liver	15.0 (M)	38.4 (M)	35.9 (M)
	8.5 (F)	20.8 (F)	20.5 (F)
Bladder	21.7 (M)	22.9 (M)	29.3 (M)
	4.2 (F)	4.3 (F)	3.1 (F)
Leukaemia	10.1 (M)	8.3 (M)	13.1 (M)
	7.5 (F)	6.7 (F)	7.8 (F)
Breast	32.4	30.3	35.6
Prostate	21.6	20.1	25.8

Despite making headlines of national newspapers, the study was not peer-reviewed and marked by limitations that could hamper ongoing research. As pointed out by other CNR researchers, the geographical boundaries and investigated cancer types were selected *a priori*, data were far too scarce to suggest a causal link and authors of previous epidemiological work had not been consulted [15, 16]. In 2004, the National Department for Civil Protection then launched a study covering a larger areas and analysing data for 20 types of cancer and 11 types of congenital malformations (CM), which included researchers from the WHO, ISS and CNR. Using an environmental waste index (EWI) to asses waste-related pollution across 196 municipalities, this multi-institutional study reported significant excess relative risks (ERR) for all-cause mortality (9.2% men, 12.4% women), all-cancer mortality and mortality from liver, lung and stomach cancer in high-EWI relative to low-EWI municipalities, as well as an 83% increase in the risk of CM of the urogenital and central nervous systems [17, 18]. Whilst authors were cautious about inferences of causality, the study showed that the highest rates of mortality and CM were found in areas with the highest concentrations of illegal landfills and poor indices of socioeconomic deprivation, including the ‘triangle’ towns of Acerra and Nola.

Reactions of regional and national health authorities to these findings were generally aimed at reassurance of local communities and did not explicitly recognise a risk to public health [16]. This stance was exemplified by the director of the Regional Health Agency, who in July 2008 criticised the multi-institutional study by claiming that uneven distribution of mortality clusters could be explained by confounding factors like smoking or viral hepatitis. Moreover, he stated that data on CMs were unsubstantiated as ∼25% of Campanian hospitals had for several years not contributed to the regional CM registry [16, 19]. The authors responded that there was no evidence that smoking or viral hepatitis could act as confounders (i.e. have similar distribution to waste-related environmental pressure) and that uneven reporting of CM would actually lead to underestimation of risk as most hospitals that did not contribute to the regional registry were found between Naples and Caserta [20, 21]. Nevertheless, the outcome of such criticisms was a focus on initiation of comprehensive long-term studies rather than short-term interventions. Since 2008, the ‘land of fires’ has thus become the site of a vast experiment in ‘exposomics’, which aims to identify biomarkers of environmental exposures during individual's lifetimes. This has included epidemiological studies analysing waste-related health outcomes as well as biomonitoring studies measuring markers of exposure to toxic waste in the population [22].

Epidemiological and biomonitoring studies on the population of the ‘land of fires’ have been the subject of systematic reviews [23, 24, 25, 26]. There is consistent evidence in support of association between proximity to toxic waste in the Naples-Caserta area and increase in pollutant exposure, cancer mortality and CMs. Quality of evidence remains limited by major heterogeneity in study design (e.g. mortality analyses, cluster analyses, correlational studies), limited adjustment for confounders and incomplete coverage—a regional cancer registry was only established in 2012.

Biomonitoring studies have observed a significant contamination of animals and humans living close to illegal landfills with dioxins and polychlorinated biphenyls (PCB). Studies on bovine milk samples from farms in Northern Campania found that about two thirds of samples had dioxin levels over the European Commission safety threshold of 3.0 pg per gram of fat and one fourth of samples had PCBs over 5.0 pg/g fat [27, 28]. Dioxin and PCB contamination was also observed by studies on milk from breastfeeding women in the Naples-Caserta area. Critically, dioxin levels (mean 16.6 pg/g fat) were positively related to the age of participants and their proximity to toxic waste disposal sites [29, 30]. These data are consistent with data from milk, blood and serum samples of over 850 participants in the SEBIOREC study, which found significant overexposure to dioxins and heavy metals (e.g. cadmium, mercury) for participants living in sites of the Naples-Caserta area with high environmental pressure due to waste [31, 32]. In 2021, finally, the region-wide SPES study from the Zooprophylactic Institute in Portici (IZSM) and National Tumour Institute in Naples, including over 4,200 participants, showed that serum samples from the ‘land of fires’ cluster (Acerra-Naples) had high levels of dioxins and PCBs relative to clusters with lower waste-related environmental pressure [33, 34, 35].

**Figure 2:**
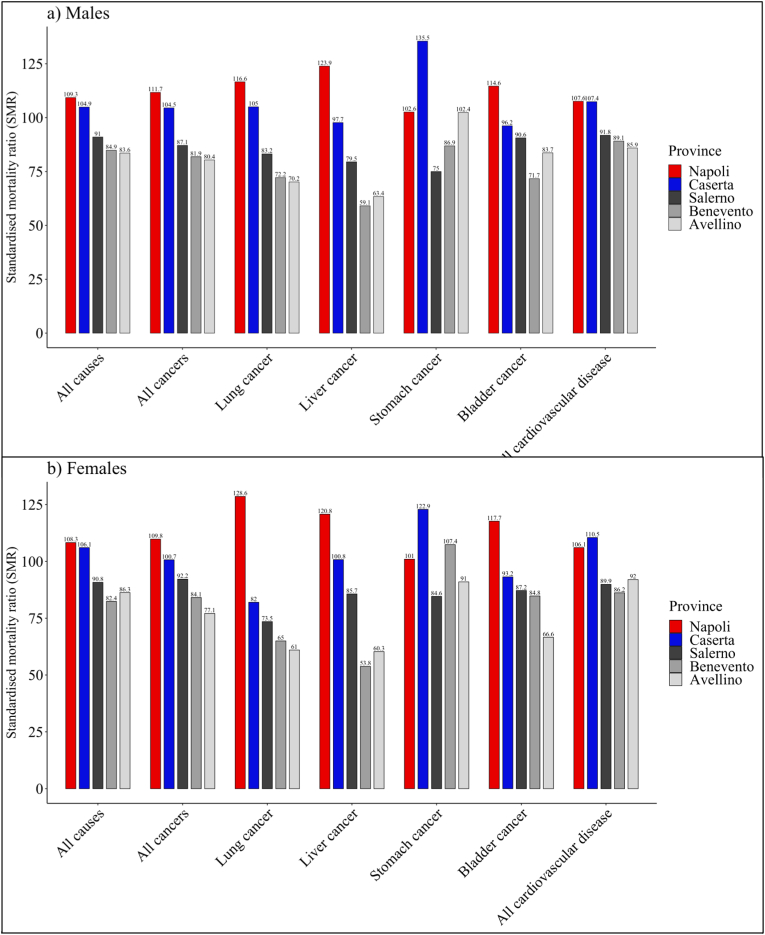
Age-adjusted SMRs in Campanian provinces (1998–2001). *Data from:* Mazza A et al. L. Illegal Dumping of Toxic Waste and Its Effect on Human Health in Campania, Italy. Int J Environ Res Public Health. 2015;12(6):6818-31.

Epidemiological studies on the population of the ‘land of fires’ in the 2000–2015 period have yielded three main conclusions. Firstly, all-cancer mortality rates in Campania were lower than national averages in the 1980s–90s but are currently higher, having only minorly benefitted from the generalised decrease in both all-cause and all-cancer mortality in the last three decades [24, 36]. Secondly, age-adjusted standardised mortality ratios (SMR) for all causes, all cancers and cardiovascular disease are significantly higher than expected in the provinces of Naples and Caserta, unlike in the other provinces of Salerno, Benevento, and Avellino (Figure 2) [37] (see Figure 2).

**Figure 3:**
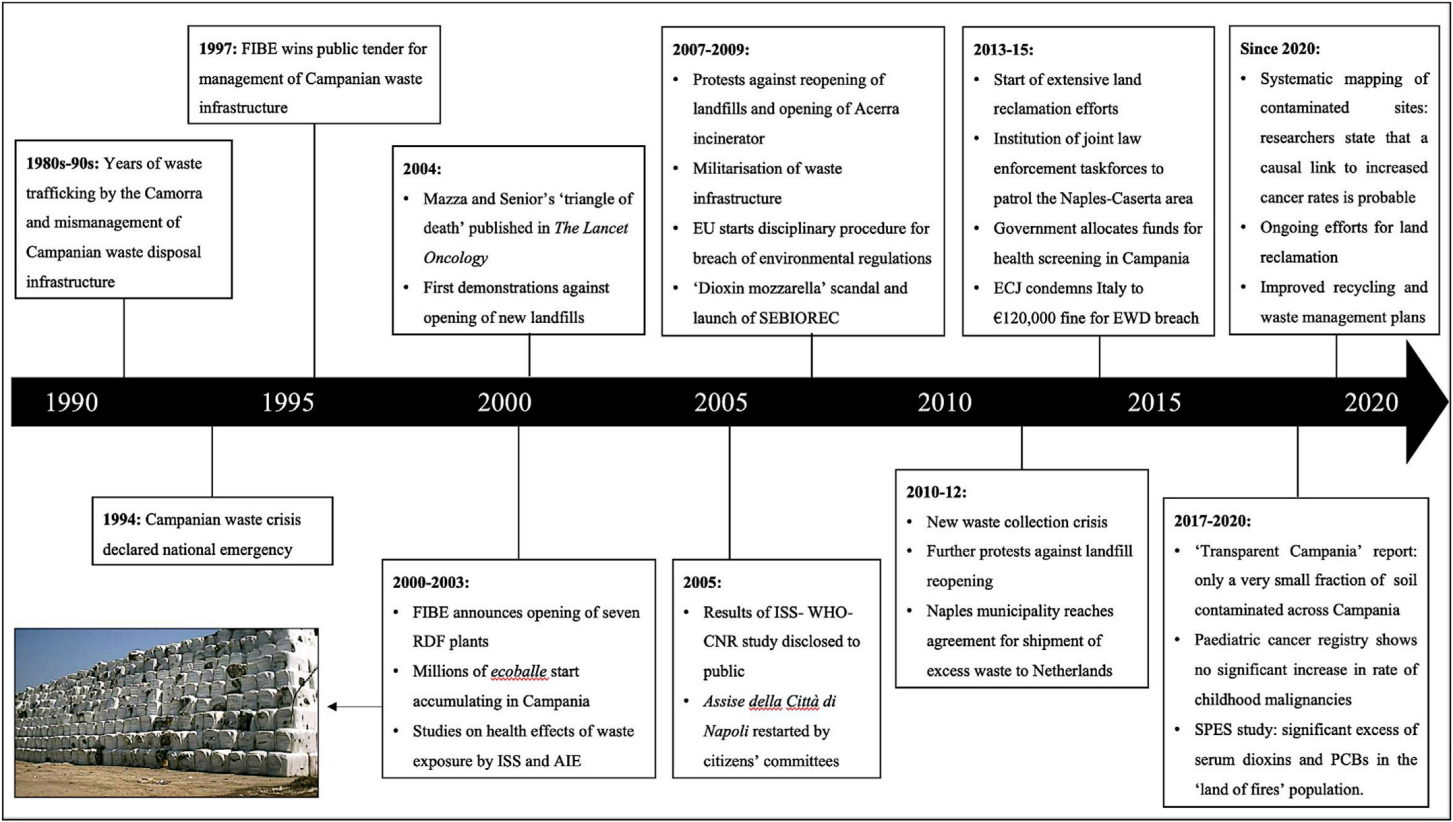
Timeline of political responses to the Campanian waste emergency. *Adapted from:* Armiero M. Is there an indigenous knowledge in the urban north? Re/inventing local knowledge and communities in the struggles over garbage and incinerators in Campania, Italy. Estudos de Sociologia. 2014;1.

Finally, correlational studies, including the Italy-wide SENTIERI study on contaminated sites of national concern, run by the ISS between 2007 and 2010, identified excesses of incidence, hospitalisation and mortality due to liver, lung, stomach and bladder cancer in the contaminated site formed by 55 municipalities across the Naples and Caserta provinces, even after adjustment for socioeconomic deprivation [38, 39, 40]. Remarkably, however, few to no studies accounted for major environmental confounders (e.g. smoking, traffic pollution, viral hepatitis) that are also likely play an important role in cancer incidence and mortality in the area [25, 26].

A more complex picture has emerged from studies on the paediatric population of the ‘land of fires’. On the one hand, studies have consistently shown a small but significant increase in the incidence of CM, mainly of the urogenital system, which is compatible with a short term effect of proximity to illegal landfills and waste burning sites [25]. On the other hand, data from the regional register of infantile and paediatric cancers in the 2008–12 period showed that cancer incidence in the 0–14 (mainly leukaemias and lymphomas) and 15–19 (mainly thyroid cancer) age cohorts was in line with national averages, with no significant differences in distribution across Campanian provinces [41, 42]. While it cannot be excluded that differences in incidence or mortality were masked by the rarity of childhood cancer, available evidence suggests that any waste-related impact on local malignancy patterns is likely limited to the adult population.

Aside from being an interesting case of ‘real world’ epidemiology, this proliferation of studies after 2008 has led to a vast epidemiological survey of the ‘land of fires’ and has made Campania the most extensively biomonitored Italian region [43]. Yet, it can also be viewed as an example of how the misperception of scientific uncertainties can undermine the precautionary principle when research findings are critical to decision-making processes in public health [44]. As stated by the authors of the multi-institutional study [Martuzzi *et al.* (2009)], central to environmental epidemiology is the principle that ‘researchers should not, while looking for incontrovertible evidence, postpone decisions or, worse, interpret absence of proof as proof of absence of risks’ [45]. Some scholars have argued that, by waiting for results of further studies before initiating measures like land reclamation, extensive screening or improved access to specialist care, local and national authorities could be seen to have failed to abide by Bradford Hill's dictum that incompleteness of evidence ‘does not confer upon us the freedom to ignore the knowledge we already have, or to postpone the action that it appears to demand at a given time’ [16, 46].

This consideration becomes especially important in light of the decade-long delay with which convincing evidence for a causal link eventually emerged [47]. The best example of this is the systematic mapping of contaminated waste disposal sites in the ‘land of fires’, commissioned in 2016 to the ISS by the North Naples prosecution service and completed in 2021. The study identified 2,767 contaminated sites in 38 municipalities of the Naples-Caserta area, 90% of which were illegal landfills or uncontrolled waste burning sites that had not been subject to reclamation efforts. 37% of inhabitants were found to live within 100 m of a contaminated site. Using a Geographic Information System (GIS) geodatabase, municipal risk indices (MRI) were computed based on type and quantity of waste, nature of disposal sites and proximity to inhabitants and the municipalities grouped into 4 classes of MRI [48]. Health outcome indices (HOI) related to diseases for which hazardous waste exposure is an established risk factor [49] were then assigned to municipalities based on hospital records, discharge summaries and local registries for cancer and CMs. Alongside excesses for all-cancer, liver, lung and bladder cancer mortality in the whole study area, regression analyses found that high-MRI municipalities had significant excesses of breast cancer mortality, leukaemia incidence, hospitalisation for asthma and prevalence of preterm birth and neonatal CMs relative to low-MRI municipalities [50, 51, 52]. Along with recommendations for land reclamation and integrated environmental surveillance, the geographical specificity of the findings prompted the authors to state for the first time that waste ‘has probably caused or concurred con-causally to the occurrence of these diseases’ [50].

### 3 Economy and the media: misplaced focus in communicating health risks

Alongside risks to health in the medium term, a concern of the population of the ‘land of fires’ as well as national media has been the risk that ongoing disposal of toxic waste may result in long-term contamination of soil, groundwater and agricultural products in a territory known in antiquity as ‘*Campania felix’* for its fertility. In March 2008, while biomonitoring studies on animals and humans in Northern Campania were being conducted, routine checks by health protection units of the gendarmerie (*Carabinieri NAS*) on samples of buffalo mozzarella from farms in the Caserta area detected dioxin levels over the EU threshold of 3.0 pg/g fat [53, 54, 55]. While later tests showed that dioxin contamination was limited to a small number of samples and in any case at a level highly unlikely to cause dangers to human health, concern over food chain contamination led some countries (e.g. South Korea, Japan, Russia) to temporarily block mozzarella imports [56]. Along with ongoing mediatisation of the waste crisis, such responses were associated with a 20%–40% fall in sales of Campanian agricultural products [57, 58]. Ecological alarm was further raised by a report of the Regional Environment Agency (ARPAC) in 2011, which identified seven contaminated ‘macro-areas’ spanning 2.7 million m^2^ with an estimated waste burden of 17.4 million m^3^, where studies on soil and groundwater samples had shown high levels of heavy metals (iron, lead, arsenic, manganese) [59]. A 2012 Legambiente report summarising investigations on waste trafficking into Campania then suggested that soil and groundwater in several sites of the Naples-Caserta area were contaminated with inorganic (e.g. fluoride, heavy metals) and organic (e.g. dichloromethane, tetrachloroethylene, toluene) pollutants, to the extent that local authorities were forced to forbid agricultural use of water wells in several municipalities belonging to the two provinces [60, 61].

Since the end of the acute phase of the waste crisis and the start of land reclamation efforts, the scale of the Campanian eco-agricultural hazard has been majorly downsized, in contrast to the increasing evidence on adverse health effects. Studies by the University of Naples Federico II and IZSM have shown that contamination of soil or groundwater with dioxins or heavy metals is limited to defined micro-areas close to urban or industrial sites, which account for only 3% of the ‘land of fires’ [62]. In 2017, the report of the ‘Transparent Campania’ initiative of the IZSM, ISS and regional health authorities concluded that only 33 out 1.3 million hectares of sampled soil were contaminated based on environmental regulations and they had already been banned from agricultural use. Moreover, only 0.02% of 30,000 sampled agricultural products were found to have levels of environmental pollutants potentially noxious to health [58, 63]. These events further highlight key discrepancies in how environmental and epidemiological knowledge can be distorted in public perception. On the one hand, studies on adverse health outcomes of toxic waste exposure in Campania built up for years with reporting often limited to science sections of local newspapers. On the other hand, one incident in 2008 was mediatised to the extent of causing an international panic with severe economic repercussions on the image of Campania as a source of agricultural products with limited grounding on available evidence.

### 4 Political reactions: local communities as producers of epidemiological knowledge

Analysing the political context during the emergency in the ‘land of fires’ provides a paradigm for how perception of environmental and epidemiological risk differs between authorities and affected communities [8, 9, 16]. The Campanian waste crisis was declared national emergency in 1994, when the combination of the Camorra's activities and years of planning delays, lack of recycling and mismanagement of waste disposal infrastructure led to saturation of regional landfills (see Figure 3 for timeline) [64]. In July 1997, the President of Campania and appointed manager of the crisis released a plan that aimed to resolve the emergency by converting excess waste into refuse-derived fuel (RDF), which was then to be burned in incinerators. The plan was tendered to FIBE, a consortium of Italian and German companies that was free to choose the sites and opening dates of waste management plants. By 2003, seven RDF production plants had been opened but no incinerators, so that RDF plants could only produce large packages of uncompacted waste (*ecoballe*) whose wet waste fraction was too high for incineration [65]. As a result, *ecoballe* were just stocked in the open and built up to 6 million tonnes by 2008, with regional authorities forced to reopen decommissioned landfills and ship excess waste to other regions and to Germany [66, 67]. In 2004, citizens started to protest authorities' perceived inaction (often violently) and to voice their opposition to opening of landfills and incinerators [16, 68]. The government issued a decree (DL 90/2008) commissioning four new incinerators and ten landfills, often found in highly socioeconomically deprived areas like the Neapolitan neighbourhoods of Pianura and Chiaiano [68]. The new landfills were militarised and protests declared acts of subversion against ‘sites of strategic national interest’ [69]. After transfer of ∼170,000 tonnes of waste to the new landfills as part of the ‘Clean Roads’ operation in 2008 and the opening of the first (and only) incinerator in Acerra in 2009, the end of the emergency was declared, despite the ongoing stockpiling of *ecoballe*^2^ [16, 70, 71, 72]. The crisis recurred in 2010, when 600 tons of waste accumulated in the streets of Naples and local inspectors of the European Commission commented that the situation was unchanged from 2008 due to the lack of an integrated regional plan for waste management and recycling [73, 74]. A solution was eventually devised in 2012, as the municipal government reached an agreement for shipment of 248,000 tonnes of waste to the Netherlands to relieve the burden of regional landfills [75]. However, quantitative studies comparing produced waste flows (urban waste, industrial waste, RDF) with intake of regional landfills in the 1999–2007 period found that ∼1.9 million tonnes of waste appear to have gone missing from official records, which has been a cause of concern in a territory with historical involvement of the eco-mafia in illegal disposal of waste [76].

Sociological studies on media reactions during the ‘land of fires’ crisis [2004-8] have observed that popular protests against opening of landfills and incinerators, in particular violent clashes with authorities, were the most publicised aspect of the emergency [16, 77]. Commentaries on national media tended to suggest that mobilisations were driven by a pre-political, ideologically driven opposition to all proposed solutions, motivated by ‘technophobia’, ‘obsessed localisms’ and a NIMBY (‘not in my backyard’) attitude that bore ‘significant responsibility in paralysis of waste management in Campania’ [68, 78, 79, 80]. This response was often paired with reluctance to acknowledge public health risks for affected communities [16]. For example, when in April 2008 authors of the multi-institutional (WHO, ISS, CNR) study issued a plea for initiation of urgent land reclamation efforts despite uncertainty of epidemiological data, the then Director of the Prevention Department in the Ministry of Health stated that a link between exposure to hazardous waste and increased cancer prevalence ‘did not exist’ and that public concerns were to be attributed to the ‘ignorance of innocent populations, incompetence of some colleagues and illegality of those profiting from the waste business’ [81].

In academic contexts, the ‘land of fires’ crisis is also remembered for the polarisation it caused within the Italian epidemiological community between scholars who tended to reassure local populations that evidence was too incomplete to hypothesise a public health risk and scholars who instead stressed the need for intervention before further studies were carried out [16, 82]. Moreover, some epidemiologists became interested in how popular mobilisations had fostered development of lay knowledge of environmental health issues within local communities. In particular, studies found that activists grounded their opposition in factual arguments, pointing out the failure of designated landfill sites to meet environmental regulations, their proximity to residential areas or previous use by the Camorra for disposal of toxic waste [16, 83]. Citizens' committees protested a provision in the 2008 governmental decree that allowed both ordinary and hazardous waste to be disposed of in new landfills, even though Italy had since 2004 been issued warnings by the European Commission for breaching the Hazardous Waste and Landfill Directives [14]. In 2010, Italy was condemned by the European Court of Justice (ECJ) for ‘not adopting measures required to avoid endangering health and the environment’ [14, 84, 85, 86]. This political context has prompted scholars to suggest that protests during the Campanian waste crisis could even be interpreted as a type of struggle for environmental justice by the population of an ‘interstitial South’ existing in the Global North but separated from it by socioeconomic inequalities, drawing parallels with environmental struggles in the Global South [16, 68]. By combining their experiences of the crisis with information from experts, activists could also be viewed as producers of a type of indigenous knowledge or ‘street science’ that allowed them to explore the uncertainty and ‘internal pluralities’ of scientific and especially epidemiological evidence [68, 87]. An example of this is the *Assise della Città di Napoli*, an assembly initiated in 1991 as a forum to discuss issues affecting local communities that was revived by academics in 2005 in the wake of the crisis. Every week, the *Assise* gathered experts (e.g. epidemiologists, environmental scientists, geologists, legal scholars) to discuss developments in the emergency, provide updates on political responses to the crisis and inform citizens' committees of research findings [68, 88]. From 2005 to 2010, the *Assise* thus acted as a ‘popular university’ for citizens involved in grassroots initiatives, to the point that many local activists became authors of informational pamphlets about key aspects of the Campanian waste emergency [68, 88, 89].

## 5 Outlook and conclusions

As a case study in the evolution of environmental emergencies, the ‘land of fires’ shows several analogies with larger crises, including climate change. It sheds light on the interdependence of academic debate, media communication, economic considerations and responses by authorities and local communities. Since 2001, researchers have issued recommendations to mitigate the Campanian emergency that can be summarised around five points [11, 50, 60].

1. Increased prosecution of illegal and uncontrolled waste disposal activities
2. Reclamation of contaminated waste disposal sites and surrounding areas
3. Improved waste infrastructure management and recycling across Campania
4. Activation of an integrated epidemiological surveillance plan for affected populations
5. Improved public health measures including prevention, early diagnosis, and specialist care

Progress has been made in legislation aimed at prosecuting illegal waste disposal. The national Waste Management Act (Dlgs 22/1997) and Environmental Code (Dlgs 152/2006) introduced the crimes of ‘organised activities for illegal waste trafficking’ and illicit waste burning, along with frameworks for tracing waste flows and reclaiming contaminated sites [60, 90, 91]. Since 2008, several illegal waste disposal sites have been seized by authorities, although experts have observed that reclamation efforts are ongoing or yet to start in most sites due to planning delays [92]. In 2013, local authorities, law enforcement agencies and environmentalist organisations reached an agreement for institution of joint taskforces to patrol the ‘land of fires’, including with surveillance drones [93, 94]. Finally, initiation of integrated regional plans for urban and industrial waste management in the last decade has led to increase in recycling of urban waste in Campania from 29.3% (2009) to 54.2% (2020) and to opening of facilities for conversion to RDF of 3.3 million tonnes of residual *ecoballe* in Campanian landfills [95, 96, 97]. Yet, Campania still sends 30% of its dry urban waste and 90% of its wet urban waste to other regions due to capacity constraints and has since 2015 been condemned by the ECJ to a daily fine of €120,000 due to its ongoing failure to comply with the European Waste Directive (EWD) [92, 95, 98].

Healthcare responses to the ‘land of fires’ crisis are less clear, and this may reflect the delay with which a possible causal link between waste exposure and health risk was acknowledged. In 2013, the government issued a dedicated decree (DL 136/2013) appropriating €25 million in yearly funds for ‘potential screening and prevention measures to promote the health of local populations’, whose nature was not specified [25, 99]. Of the three cancer types with screening programs of proven efficacy (breast, cervix, colorectal), only breast cancer has been linked to toxic waste exposure in Campania and there has been no suggestion to expand mammography services to the whole local or regional population [25]. While the decree may be referring to biomonitoring, only two large-scale human biomonitoring studies have been conducted so far (SEBIOREC, SPES) and evidence that data on individuals' exposomic profile could be usefully integrated into cancer risk prediction algorithms is very limited [25, 32, 35, 100]. By contrast, completion of regional and local registries for cancer and CMs in the last decade has clearly led to more integrated epidemiological surveillance of the Campanian population. However, recent attempts to translate this into improved early prevention efforts in local health services were delayed by the COVID-19 pandemic. Furthermore, illegal waste burning in the Naples-Caserta territory continued—albeit at reduced intensity—despite the decrease in waste production flows associated with lockdown measures in 2020 and 2021 [101]. In coming years, coordinated interventions by regional healthcare services and national public health authorities may help further elucidate epidemiological risks in the ‘land of fires’ and thereby support implementation of preventative strategies in favour of communities affected by the waste emergency in the last three decades.

## Declarations

### Author contribution statement

All authors listed have significantly contributed to the development and the writing of this article.

### Funding statement

This research did not receive any specific grant from funding agencies in the public, commercial, or not-for-profit sectors.

### Data availability statement

Data included in article/supp. material/referenced in article.

### Declaration of interest’s statement

The authors declare no competing interests.

### Additional information

No additional information is available for this paper.

## Footnotes

1 The term refers to fires lit by the Camorra to dispose of waste in illegal landfills and was introduced in 2003 by environmentalist organisation Legambiente to describe the area of action of the Campanian eco-mafia.

2 The Acerra incinerator remains unsuitable for burning of *ecoballe*-derived RDF and to this day only burns ordinary solid waste, which has in some years been associated with emissions of particulate matter (PM_10_) in excess of environmental regulations [72].
